# Impact of tissue transport on PET hypoxia quantification in pancreatic tumours

**DOI:** 10.1186/s13550-017-0347-3

**Published:** 2017-12-22

**Authors:** Edward Taylor, Jennifer Gottwald, Ivan Yeung, Harald Keller, Michael Milosevic, Neesha C. Dhani, Iram Siddiqui, David W. Hedley, David A. Jaffray

**Affiliations:** 1 Princess Margaret Cancer Centre, University Health Network, Toronto, Canada; 2 Techna Institute, University Health Network, Toronto, Canada; 3 Department of Medical Biophysics, University of Toronto, Toronto, Canada; 4 Department of Radiation Oncology, University of Toronto, Toronto, Canada; 5 Division of Medical Oncology and Hematology, Princess Margaret Cancer Centre, Toronto, Canada; 6 Department of Pathology, Hospital for Sick Children, Toronto, Canada; 7 Institute for Biomaterials and Biomedical Engineering, University of Toronto, Toronto, Canada

**Keywords:** Positron emission tomography, Hypoxia imaging, PET tracer kinetic modelling

## Abstract

**Background:**

The clinical impact of hypoxia in solid tumours is indisputable and yet questions about the sensitivity of hypoxia-PET imaging have impeded its uptake into routine clinical practice. Notably, the binding rate of hypoxia-sensitive PET tracers is slow, comparable to the rate of diffusive equilibration in some tissue types, including mucinous and necrotic tissue. This means that tracer uptake on the scale of a PET imaging voxel—large enough to include such tissue *and* hypoxic cells—can be as much determined by tissue transport properties as it is by hypoxia. Dynamic PET imaging of 20 patients with pancreatic ductal adenocarcinoma was used to assess the impact of transport on surrogate metrics of hypoxia: the tumour-to-blood ratio [TBR(*t*)] at time *t* post-tracer injection and the trapping rate *k*_3_ inferred from a two-tissue compartment model. Transport quantities obtained from this model included the vascular influx and efflux rate coefficients, *k*_1_ and *k*_2_, and the distribution volume *v*_*d*_≡*k*_1_/(*k*_2_+*k*_3_).

**Results:**

Correlations between voxel- and whole tumour-scale *k*_3_ and TBR values were weak to modest: the population average of the Pearson correlation coefficients (*r*) between voxel-scale *k*_3_ and TBR (1 h) [TBR(2 h)] values was 0.10 [0.01] in the 20 patients, while the correlation between tumour-scale *k*_3_ and TBR(2 h) values was 0.58. Using Patlak’s formula to correct uptake for the distribution volume, correlations became strong (*r*=0.80[0.52] and *r*=0.93, respectively). The distribution volume was substantially below unity for a large fraction of tumours studied, with *v*_*d*_ ranging from 0.68 to 1 (population average, 0.85). Surprisingly, *k*_3_ values were strongly correlated with *v*_*d*_ in all patients. A model was proposed to explain this in which *k*_3_ is a combination of the hypoxia-sensitive tracer binding rate *k*_b_ and the rate *k*_eq_ of equilibration in slow-equilibrating regions occupying a volume fraction 1−*v*_*d*_ of the imaged tissue. This model was used to calculate the proposed hypoxia surrogate marker *k*_b_.

**Conclusions:**

Hypoxia-sensitive PET tracers are slow to reach diffusive equilibrium in a substantial fraction of pancreatic tumours, confounding quantification of hypoxia using both static (TBR) and dynamic (*k*_3_) PET imaging. TBR is reduced by distribution volume effects and *k*_3_ is enhanced by slow equilibration. We proposed a novel model to quantify tissue transport properties and hypoxia-sensitive tracer binding in order to improve the sensitivity of hypoxia-PET imaging.

**Electronic supplementary material:**

The online version of this article (doi:10.1186/s13550-017-0347-3) contains supplementary material, which is available to authorized users.

## Background

Positron emission tomography imaging of hypoxia is a promising way to detect hypoxia non-invasively in solid tumours [1, 2]. A major challenge to this approach is that the binding rate of hypoxia-sensitive PET tracers such as fluoromisonidazole (FMISO) and fluoroazomycinarabinoside (FAZA) is slow as compared to, e.g., flurodeoxyglucose (FDG), and can be comparable to diffusive equilibration rates in tumour tissues.

As an example, a typical threshold used to decide whether or not a PET voxel hypoxic is that the voxel-scale tracer concentration exceeds that in blood by 20% after 2 h; i.e.,TBR (2 h) >1.2 [3–5]. This means that the binding rate of tracer in hypoxic tissue is

1 $$ k_{\text{b}}\gtrsim \frac{0.2}{2\mathrm{ h}} = 0.1~\mathrm{h}^{-1}.  $$

In comparison, the rate at which tracer diffuses across a distance *l* through the extravascular space of tissue scales as

2 $$ k_{\text{eq}} \sim D/l^{2},  $$

where *D* is the diffusivity of the tracer. For FAZA and similarly sized molecules (on the order of several hundred Daltons), *D*∼10 *μ*m^2^/s in most tissue [6, 7]. Hence, taking *l*∼100 *μ*m to be the distance between capillaries, the equilibration rate *k*_eq_∼20 h^−1^ for tracer is typically much faster than the binding rate, and comparable to the rate of extravasation, *k*_1_.

On the other hand, for tissue with substantial mucous deposits (common in carcinomas [8] such as pancreatic ductal adenocarcinoma [9]), where diffusivity can be slowed by two or more orders of magnitude [10, 11], the rate of equilibration slows drastically, becoming comparable to the binding rate. This can also happen in tissue with necrotic regions ($l\gtrsim 500\;\mu \mathrm {m}$) interspersed with hypoxic cells.

Slow diffusive equilibration has two important consequences for quantifying tumour hypoxia based on tracer uptake. First, if an imaging voxel contains both hypoxic cells and either mucous or small necroses, the voxel-scale TBR value will be reduced by the fact that tracer does not reach diffusive equilibrium at the standard imaging time, between 2 and 3 h post-injection. Hence, the sensitivity of static PET imaging to hypoxia is diminished. Second, as tracer slowly equilibrates in mucinous and necrotic tissue, its concentration increases at a rate comparable to that due to hypoxia-induced binding and a compartment model [12–15] may not be able to distinguish the two processes. In this case, we hypothesize that the trapping rate *k*_3_ represents a sum of the binding rate *k*_b_ and the rate of equilibration. Quantifying hypoxia based on *k*_3_ will thus overestimate its extent since *k*_3_≥*k*_b_.

In this paper, we seek to test these hypotheses by modeling the pharmamcokinetics of FAZA in 20 patients with pancreatic ductal adenocarcinoma (PDAC), applying basic principles of diffusive equilibration to interpret transport data calculated from a standard two-tissue compartment model.

## Methods

### Patient population and PET/CT scans

Data was taken from 20 patients with biopsy-confirmed pancreatic ductal adenocarcinoma and FAZA-PET scans. Dynamic PET imaging scans were acquired over 1 h following injection of FAZA. The 1-h time-activity curves (TAC _1_) were each binned into 34 frames: 12 10-s frames, followed by 8 32-s frames, followed by 7 2-min frames, followed by 7 5-min frames. Patients returned for a static PET scan at 2 h. CT scans used for co-registration were taken at the beginning of the dynamic and static PET scans. Further details of this patient cohort and the PET/CT scans have been described previously [16].

### Region of interest contours

PET activity data was obtained for regions of interest (ROIs) contoured using co-registered CT images. Tumour ROIs were contoured by a radiologist using the CT scan at 2 h. This was co-registered manually to the initial CT scan and the two CT ROI sets were co-registered to the dynamic and static PET scans. In order to minimize effects resulting from high liver uptake of FAZA, aorta ROIs were contoured from the same range of PET/CT slices (along the cranial-caudal axis) as the tumour ROIs. At the level of the pancreas, the aorta is between 1.5 and 2 cm in diameter; to minimize partial volume effects, ROIs in the aorta were restricted to 0.75 cm in diameter and combined so that at least 25 PET voxels (3.9 ×3.9×3.3 mm ^3^ each) were imaged.

### Compartment model analysis

Dynamic PET TACs of FAZA were analyzed using the two-tissue compartment model [12–15, 17–19]:

3 $$ \frac{d C_{d}(t)}{dt} = k_{1}C_{\text{In}}(t)-\left[k_{2}+k_{3}\right]C_{d}(t)  $$

and

4 $$ \frac{dC_{b}(t)}{dt} = k_{3}C_{d}(t).  $$

Here, the concentration of tracer in the extravascular space of an imaged region has been partitioned into an unbound, diffusing component *C*_*d*_ as well as a component *C*_*b*_ that is bound by hypoxia. *C*_In_ is the “input” function, which we took to be the imaged tracer concentration in the aorta, as described above. As noted earlier, *k*_1_ and *k*_2_ are the vascular influx and efflux coefficients and *k*_3_ is the tracer trapping rate. The total tracer concentration in an imaged region is

5 $$ C(t) = v_{b}C_{\text{In}}(t) + (1-v_{b})\left[C_{d}(t)+C_{b}(t)\right],  $$

where *v*_*b*_ is the volume fraction occupied by blood in the region of interest.

The above model was fitted to both the 1-h TACs (TAC _1_) as well as the combined 2-h TACs (TAC _2_) comprising the 1-h TACs plus static scans at 2 h (in part to asses co-registration errors, which should be greater for TAC _2_). Coefficients (*v*_*b*_, *k*_1_, *k*_2_, and *k*_3_) were determined by minimizing

6 $$ \chi^{2} = \sum_{i}^{N}w_{i}\left[C_{\text{model}}(t_{i})-C_{\text{data}}(t_{i})\right]^{2},  $$

where *C*_model_(*t*_*i*_) are the model activity values [Eqs. (3)–(5)] and *C*_data_(*t*_*i*_) are the measured values acquired during the *N* discrete time frames; *N*=34 for TAC _1_ and *N*=35 for TAC _2_. To avoid over-weighting short-duration early time frames, we used the weighting function *w*_*i*_=*δ* *t*_*i*_ in Eq. 6, where *δ* *t*_*i*_ was the duration of the *i*th time frame (because the *t*=2 h time-point in TAC _2_ did not represent a true 1-h time bin beyond the TAC _1_ data set, we used *δ* *t*_35_=*δ* *t*_34_=5 min). Equation 6 was minimized in Wolfram Mathematica 11.1 using its built-in numerical minimization routine (NMinimize) with *C*_model_(*t*_*i*_) calculated using trapezoidal integration.

An important tissue transport quantity is the *distribution volume*:

7 $$ v_{d} \equiv \frac{k_{1}}{k_{2}+k_{3}}.  $$

It represents the volume fraction of an imaged ROI in which tracer initially fills; i.e., rapidly equilibrates in. Patlak’s formula [20, 21],

8 $$ \text{TBR}(t) = v_{b} + (1-v_{b})v_{d} + K_{i} (1-v_{b})\frac{\int^{t}_{0}\;d\tau\;C_{\text{In}}(\tau)}{C_{\text{In}}(t)},  $$

for the tumour-to-blood ratio at time *t* was used to “correct” TBR for distribution volume effects:

9 $$ \begin{aligned} \text{TBR}_{\text{corrected}}(t) & \equiv \frac{\text{TBR}(t)-v_{b}(1-v_{d})}{v_{d}} \\ & = 1 + k_{3}(1-v_{b})\frac{\int^{t}_{0}\;d\tau\;C_{\text{In}}(\tau)}{C_{\text{In}}(t)}. \end{aligned}  $$

In Eq. (8), *K*_*i*_≡*k*_3_ *v*_*d*_ is sometimes referred to as the “net trapping rate”. TBR _corrected_ represents the theoretical tumour-to-blood ratio that would have arisen had the distribution volume been unity.

Correlations were analyzed between *k*_3_, *v*_*d*_, TBR, and TBR _corrected_, where TBR was calculated as

10 $$ \text{TBR}(t) \equiv \frac{C_{\text{data}}(t)}{C_{\text{In}}(t)}  $$

at both *t*=1 and 2 h. Pearson correlation coefficients were calculated to quantify correlations between voxel- and tumour-scale values of these quantities. Voxel-scale coefficients were calculated by fitting the above model to the individual TACs for each voxel, while tumour-scale values were obtained using the average TAC in each tumour. Correlations were reported as the population average (over twenty tumours) of the intra-tumour voxel-scale *r* values (“voxel-scale”) and as correlations between tumour-scale values (“tumour-scale”).

## Results

### Correlations between TBR and *k*_3_

**Fig. 1:**
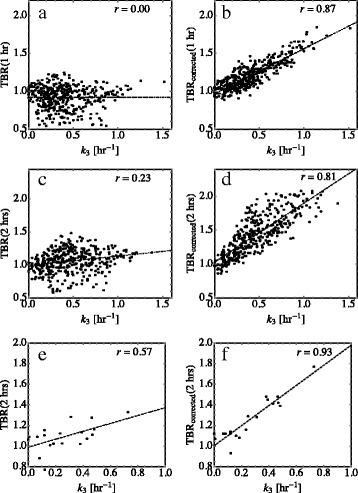
Correlations between tumour-to-blood uptake ratios and the trapping rate are enhanced when uptake is corrected for the distribution volume. Left side: tumour-to-blood uptake ratio of FAZA versus trapping rate; right: tumour-to-blood uptake ratio corrected for the distribution volume versus trapping rate. **a** and **b** voxel-scale values for a representative patient tumour (pt. 2) using TAC _1_. **c** and **d** same as **a** and **b** but with TAC _2_. **e** and **f** Tumour-scale values using TAC _2_ for all 20 tumours. Pearson correlation coefficients are shown

Comparing voxel-scale *k*_3_ and TBR values in each tumour, weak correlations were found at 1 h (average of voxel-scale *r* values = 0.10) and at 2 h (*r* value = 0.01). Patient-specific results are shown in Online Resource 1 (Additional file 1). Strong correlations were found between voxel-scale *k*_3_ and TBR_corrected_ at 1 h (population average *r* value = 0.80) and moderate correlations were found at 2 h (*r* value = 0.53). Although standard imaging protocols call for measurement of TBR at least 2 h after tracer injection, transport coefficient (*v*_*b*_, *k*_1_, *k*_2_, *k*_3_) values obtained using the 1- and 2-h data sets were equivalent to within fit errors to the compartment model. The reduction in correlations is thus a metric for co-registration errors between the 1- and 2-h data sets, as well as the diminished validity of Eq. (8), which is only a good approximation at times less than the equilibration time 1/*k*_eq_ [21]. Representative voxel-scale correlations are shown in Figs. 1a–d for one patient. Table 1 displays population averages of voxel-scale correlations using the 2-h data sets as well as the mean values of the corresponding quantities.

**Table 1:** Top: Correlation matrix of Pearson correlation coefficients between the mean voxel-scale parameters across the twenty tumours studied using the 2-h data sets. Bottom: Population average values of the corresponding voxel-scale coefficients. Standard deviations of mean values across patients are indicated in parentheses

	*k*_3_[h^−1^]	*v*_*d*_	TBR	TBR _corrected_
*k*_3_	−	*−0.59*	0.01	*0.52*
*v*_*d*_	*−0.59*	−	0.35	*−0.58*
TBR	0.01	0.35	−	0.50
TBR _corrected_	*0.52*	*−0.58*	0.50	−
	*k*_3_ [h ^−1^]	*v*_*d*_	TBR	TBR_corrected_
	0.30 (0.20)	0.85 (0.10)	1.06 (0.13)	1.25 (0.20)

Whole-tumour kinetics are less sensitive to co-registration errors and tumour-scale trapping rate exhibited modest correlations with TBR (across twenty patients, mean *r* = 0.58) but strong correlations with TBR_corrected_ (mean *r* = 0.93); see Fig. 1e, f and Table 2. Mean tumour-scale values of *k*_3_, *v*_*d*_, TBR, and TBR_corrected_ were identical to the values shown in Table 1 to within a few percent.

**Table 2:** Correlation matrix of Pearson correlation coefficients between the tumour-scale parameters across the twenty tumours studied using the 2-h data sets

	*k*_3_	*v*_*d*_	TBR	TBR _corrected_
*k*_3_	−	−0.34	*0.58*	*0.93*
*v*_*d*_	−0.34	−	0.30	−0.26
TBR	*0.58*	−0.26	−	*0.66*
TBR _corrected_	*0.93*	−0.26	*0.66*	−

### Relationship between *v*_*d*_ and *k*_3_

**Fig. 2:**
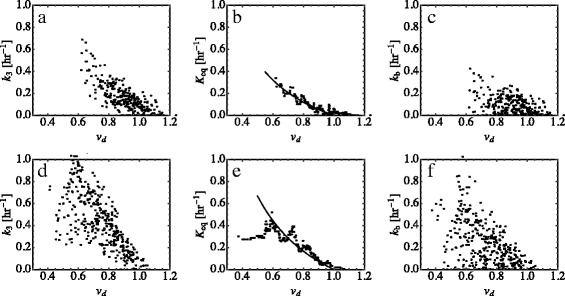
Dependence of the trapping rate on tracer equilibration and binding. **a** and **d** show voxel-scale trapping rate values versus voxel-scale TBR values for patients 1 and 2, respectively. **b** and **e** show the corresponding equilibration rates, calculated from Eq. (19); the solid lines indicate fits to Eq. (18), yielding *k*_eq_=0.45 h ^−1^ for pt. 1 and *k*_eq_=0.52 h ^−1^ for pt. 2. (**c**) and (**f**): The voxel-scale binding rates *k*_b_ calculated from Eq. (17) using the *K*_eq_ values shown in **b** and **e**

**Fig. 3:**
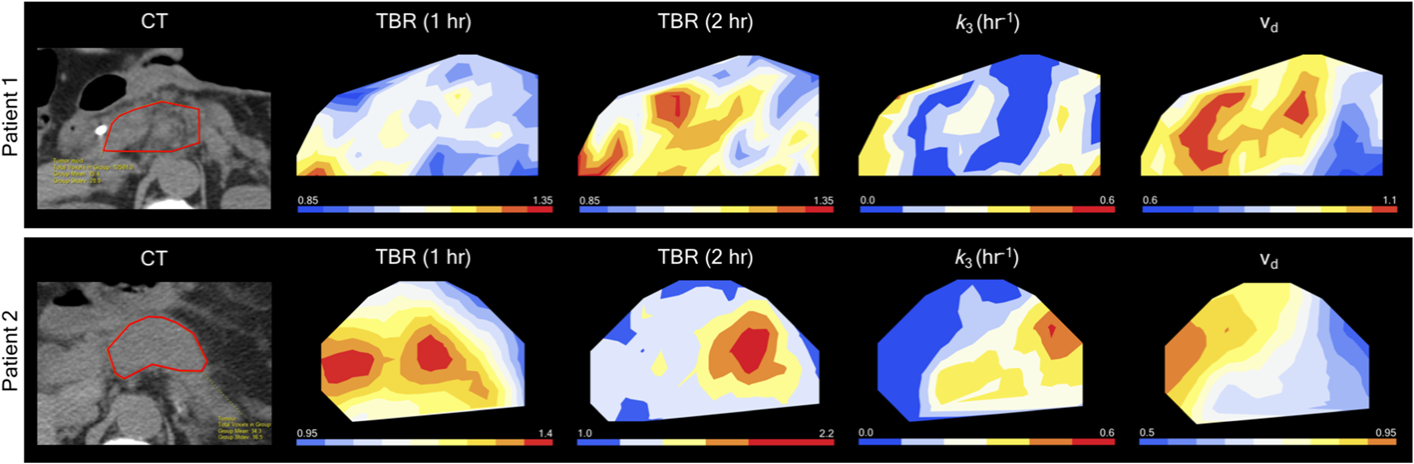
Examples of negative correlations between *k*_3_ and *v*_*d*_ and discordance between *k*_3_ and TBR in parametric maps for patients 1 and 2. From left to right: pre-PET transverse CT scan; FAZA-PET TBR at 1 h for the tumour contour shown on the CT; TBR at 2 h; *k*_3_ map; *v*_*d*_ map. Strong negative correlations between *k*_3_ and *v*_*d*_ are evident. In both tumour slices, there are regions where *v*_*d*_ is well-below unity and variations in *k*_3_ and TBR are discordant

In all patients, voxel-scale *k*_3_ values were found to depend strongly on *v*_*d*_ (population average of voxel-scale *r*-values = -0.59; see Table 1), with *k*_3_ increasing as *v*_*d*_ decreases. Figures 2a and d show two representative examples. Parametric maps of a transverse slice in each of these patients are shown in Fig. 3. Tumour-scale correlations between *v*_*d*_ and *k*_3_ are reduced (*r*=−0.34) but still substantial; see Table 2.

**Fig. 4:**
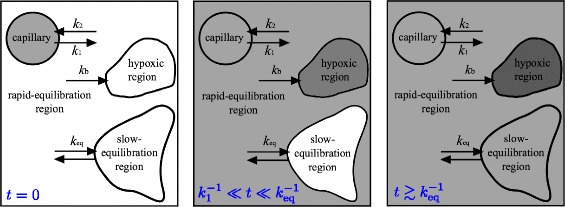
Schematic of our partitioning model. From left to right: at *t*=0 (left panel), tracer (gray-filled regions) is only in the capillary; for $k^{-1}_{1}\ll t\ll k_{\text {eq}}^{-1}$ (middle panel), tracer fills the rapid-equilibration regions and begins to bind where hypoxia arises; for $t\gtrsim k_{\text {eq}}^{-1}$ (right panel), tracer fills all regions, including the slow-equilibration regions that occupy a volume fraction *v*_*s*_ of the region of interest

To account for the unexpected correlations between *k*_3_ and *v*_*d*_, we propose a model (shown schematically in Fig. 4) in which an imaged voxel is comprised of two tissue types: one in which tracer reaches diffusive equilibration rapidly (with concentration *C* ^(*r*)^), and one in which it reaches equilibrium slowly (with concentration *C* ^(*s*)^):

11 $$ C_{d}(t) =v_{s} C^{(s)}_{d} + (1-v_{s})C^{(r)}_{d}(t).  $$

Here, *v*_*s*_ represents the voxel volume fraction in which tracer is slow to equilibrate. As noted in the Introduction, tracer will equilibrate slowly in mucinous and necrotic tissue owing to the slow diffusivity and long diffusive distances, respectively.

Having defined the above sub-compartments, the distributed-parameter compartment model [22] that describes the effects of having regions of slow-equilibration is

12 $$ \begin{aligned} \frac{d C^{(r)}_{d}(t)}{dt} &= \frac{k_{1}}{1-v_{s}}\left[C_{\text{In}}(t)-C^{(r)}_{d}(t)\right] \\ & \quad -\left(k_{\mathrm{b}}+\frac{k_{\text{eq}} v_{s}}{1-v_{s}}\right)C^{(r)}_{d}(t) + \frac{k_{\text{eq}} v_{s}}{1-v_{s}}C^{(s)}_{d}(t), \end{aligned}  $$

13 $$ \frac{dC^{(s)}_{d}(t)}{dt} = k_{\text{eq}} \left[C^{(r)}_{d}(t)-C^{(s)}_{d}(t)\right],  $$

and

14 $$ \frac{dC_{b}(t)}{dt} = k_{\mathrm{b}} C^{(r)}_{d}(t).  $$

The factors of 1−*v*_*s*_ and *v*_*s*_ here ensure detailed balance amongst the compartments. *k*_b_ is the binding rate due to hypoxia and *k*_eq_ represents the equilibration rate in the regions of slow-equilibration. Recall from the Introduction that we expect it to be on the order of (0.1→1) h ^−1^ when equilibration is driven by diffusion; see Eq. (2). In writing Eq. (14), it has been assumed that tracer does not bind inside regions of slow-equilibration since, e.g., necrotic cells and extracellular mucous deposits do not bind hypoxia-PET nitroimidazole tracers [12].

At times $k^{-1}_{1}\lesssim t\ll k_{\text {eq}}^{-1}$, after diffusive equilibration is achieved in the rapidly equilibrating regions $\left [C^{(r)}_{d}(t)\simeq C_{\text {In}}(t)\right ]$ but not yet in the slow-equilibrating regions, the tissue-to-blood ratio is readily obtained by integrating Eqs. (12)–(14):

15 $$ {\begin{aligned} \text{TBR}(t) & \simeq \;v_{b} + (1-v_{b})(1-v_{s})\\ & \quad + \left(k_{\mathrm{b}} +\frac{k_{\text{eq}} v_{s}}{1-v_{s}}\right) (1-v_{b})(1-v_{s})\frac{\int^{t}_{0} d\tau \; C^{(r)}_{d}(\tau)}{C_{\text{In}}(t)}. \end{aligned}}  $$

In arriving at this result, we have neglected back-flux from the slow-diffusion region, dropping the contribution arising from $C^{(s)}_{d}$ in Eq. (13). This is valid as long as $t\lesssim k_{\text {eq}}^{-1}$.

Since $C^{(r)}_{d}(t)\to C_{\text {In}}(t)$ for $t\gtrsim k^{-1}_{1}$, Eq. (15) is identical to the Patlak result Eq. (8), with

16 $$ v_{s} = 1-v_{d}.  $$

and

17 $$ k_{3} = k_{\mathrm{b}} + \frac{k_{\text{eq}} \left(1-v_{d}\right)}{v_{d}}\equiv k_{\mathrm{b}} + K_{\text{eq}}(v_{d}),  $$

where we have defined

18 $$ K_{\text{eq}}(v_{d}) \equiv k_{\text{eq}} (1-v_{d})/v_{d}.  $$

Equations (16) and (17) are our main theoretical results. They show that the distribution volume *v*_*d*_ defined in Eq. (7) is the volume fraction of tissue in which tracer rapidly equilibrates and that the standard two-tissue compartment model trapping rate in general represents the sum of the rate of binding due to hypoxia and the equilibration rate. In turn, this means that it is not possible to distinguish binding from equilibration from just the shape of the time-activity curves.

To distinguish *k*_b_ and *K*_eq_ in *k*_3_, voxel-scale *k*_3_ values were arranged into bins based on distribution volume values. Because there will always be a cohort of normoxic voxels in a tumour for which *k*_b_=0 (unless the hypoxic fraction is unity, simple Poissonian statistics dictates as much), it is assumed that the lowest *M* values of *k*_3_ in these bins represent equilibration:

19 $$ K_{\text{eq}} \left[(v_{d})_{i}\right] = \frac{1}{M}\sum_{j=1}^{M} \text{min}\left[\{k_{3}\}_{(v_{d})_{i}}\right]_{j}.  $$

Equation (19) is strictly valid in the limit where the variance in *k*_eq_ values is much smaller than the variance in *k*_b_ values (so that the two distributions can be distinguished). The choice of *M* is dictated by their relative sizes:

20 $$ \frac{M}{N_{b}} = \frac{\left(\left.\sigma_{k_{\text{eq}}}\right/k_{\text{eq}}\right)}{\sqrt{\left(\left.\sigma_{k_{\text{eq}}}\right/k_{\text{eq}}\right)^{2} + \left(\left.\sigma_{k_{\mathrm{b}}}\right/k_{\mathrm{b}}\right)^{2}}},  $$

where *N*_*b*_ is the total number of values within each bin, *σ*_*X*_ and *X* denote the standard deviation and mean values of *X*=*k*_b_ or *k*_eq_. Assuming that the relative variance $\left (\left.\sigma _{k_{\mathrm {b}}}\right /k_{\mathrm {b}}\right)$ is equal to that for the oxygen partial pressure $P_{O_{2}}$ (the case, e.g., when the two are related by a Michaelis-Menten-type relation [12]), the variance in *k*_b_ is expected to be large, based on the broad distribution of $P_{O_{2}}$ levels in tumours: $\left (\left.\sigma _{P_{O_{2}}}\right /P_{O_{2}}\right)\gtrsim 1$ [23]. In contrast, the relative variance in *k*_eq_—reflecting that of the size *l* of the regions in which tracer is slow to equilibrate—is small. This was estimated by calculating the variance in the minimum *k*_3_ value in each bin with respect to a *v*_*d*_-dependent average (see, e.g., the curve fits in Fig. 2). Across our twenty patients, we found an average value $\left (\left.\sigma _{k_{\text {eq}}}\right /k_{\text {eq}}\right)\sim 0.4$. As a compromise to having a sufficient number of voxels to ensure the validity of statistics and few enough to have sufficient resolution in *v*_*d*_-space to carry out these curve fits, bins were chosen to contain ten voxels. Hence, we chose *M*=0.4×10=4. A sensitivity analysis of the predicted equilibration rates and the choice of *M* is presented in Online Resource 2 (Additional file 2).

**Fig. 5:**
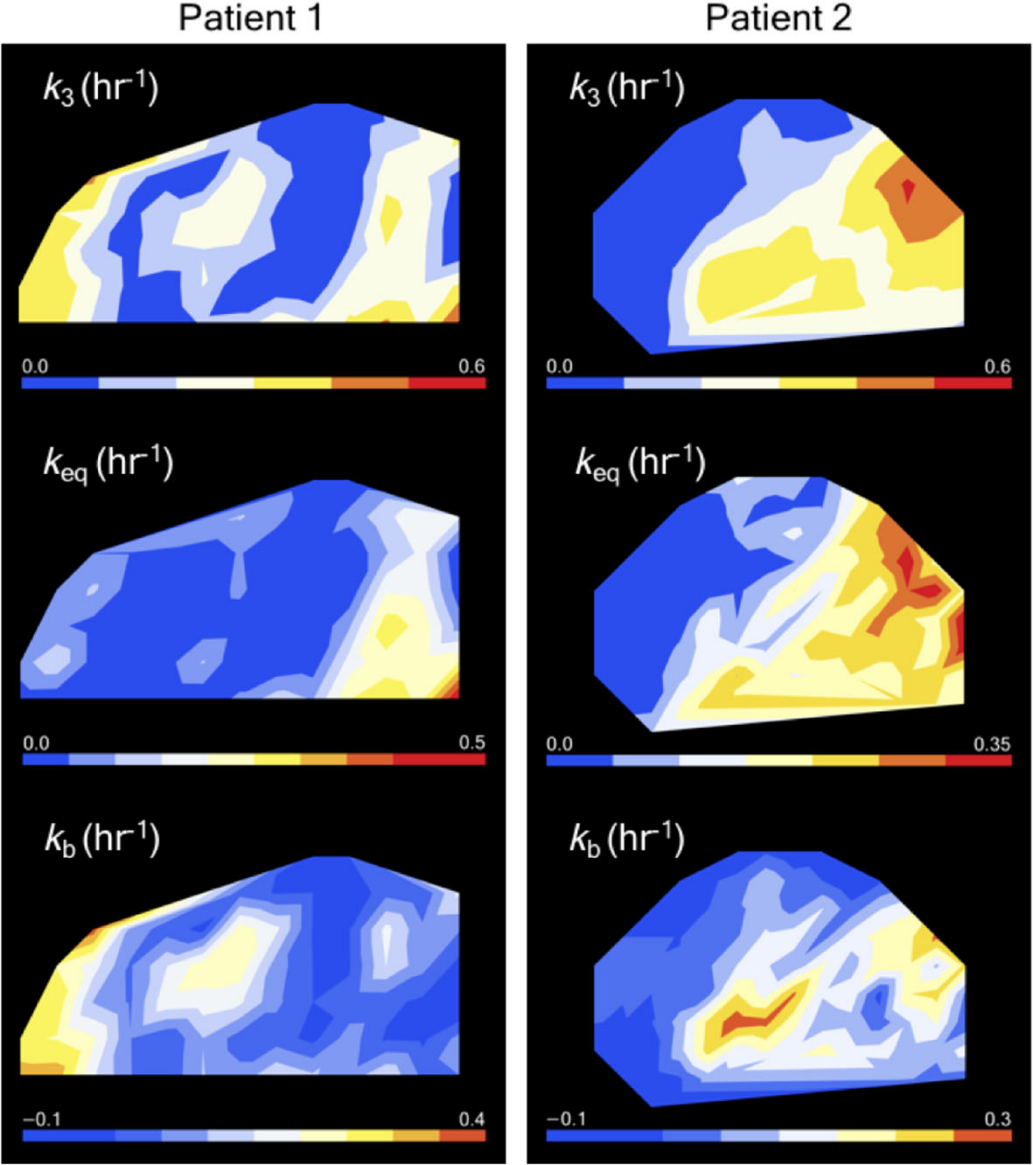
Parametric maps for an axial tumour slice from patients 1 (left) and 2 (right) showing the spatial distribution of binding and equilibration rates

An example of this algorithm is shown for two patients in Figs. 2 and 5. Voxel-scale values of *K*_eq_ in each of these bins as determined by Eq. (19) are plotted in Fig. 2b and e. The solid lines in this figure are fits to *K*_eq_(*v*_*d*_)=*k*_eq_(1−*v*_*d*_)/*v*_*d*_. (The poor fit in Fig. 2e for *λ*≲0.6 may be due to a percolation effect: for distribution volumes less than ∼0.65, regions of slow equilibration begin to overlap [24] and *v*_*d*_ will become dependent on the mean size *l* of these regions. Hence, from Eq. (2), *k*_eq_ will also begin to depend on *v*_*d*_). Also shown in Fig. 2c and f are the voxel-scale binding rates determined from Eqs. (17) and (19). Figure 5 shows parametric maps of *k*_3_, *k*_eq_ and *k*_b_ for the same tumour slices shown in Fig. 3.

The correlation matrix between derived voxel-scale parameters from our model is shown in Table 3 along with population averages of these parameters. The relative sizes of the correlations between *k*_3_ and *K*_eq_ (*r*=0.57) and *k*_b_ (*r* = 0.86) are measures of how much equilibration and binding were found to contribute to the net trapping rate *k*_3_. Most of the *v*_*d*_ dependence of *k*_3_ is contained in *K*_eq_, as evidenced by the strong correlations between *v*_*d*_ and *K*_eq_(r=−0.73) but comparatively weak correlations *k*_b_ and *v*_*d*_(*r*=−0.27). Not shown are correlations between these quantities and the vascular influx rate *k*_1_ since these were small (|*r*|<0.15) for all cases.

**Table 3:** Top: Correlation matrix of Pearson correlation coefficients between the mean voxel-scale parameters across the twenty tumours studied using the 2-h data sets. Bottom: Population-averages of the corresponding voxel-scale rate coefficients; values are shown in units of h ^−1^. Standard deviations of mean values across patients are indicated in parentheses. Also shown is the population average *k*_eq_ value, which was calculated from fits to data from all voxels in each tumour, as described in the text

	*k*_3_	*K*_eq_	*k*_b_	*v*_*d*_
*k*_3_	−	*0.57*	*0.86*	*−0.59*
*K*_eq_	*0.57*	−	0.18	*−0.73*
*k*_b_	*0.86*	0.18	−	−0.27
*v*_*d*_	*−0.59*	*−0.73*	−0.27	−
	*k*_3_	*K*_eq_	*k*_b_	*k*_eq_
	0.30 (0.20)	0.17 (0.15)	0.14 (0.08)	0.44 (0.29)

The *v*_*d*_-dependence of *k*_3_ in our model is a consequence only of mass conservation and the assumption that there exists a compartment in which tracer is slow to reach diffusive equilibrium. It does not depend on a specific microscopic model for equilibration. We tested the prediction given by Eq. (17) by fitting the binned *K*_eq_ values to a function of the form *K*_eq_(*v*_*d*_,*γ*)=*k*_eq_[(1−*v*_*d*_)/*v*_*d*_]^*γ*^ to determine how close *γ* was to its predicted value of unity. Averaging over all tumours, we found *γ*=(0.9±0.4), with the error given by the standard deviation of values across all tumours. This confirms that our model in which tracer equilibrates slowly in a fraction 1−*v*_*d*_ of tissue is consistent with our data. The mean equilibration rate derived from these fits was *k*_eq_=0.44 h ^−1^ (standard deviation of 0.29 h ^−1^ across all patients), corresponding to an equilibration time of 1/*k*_eq_∼2.3 h.

## Discussion

It is well-appreciated that the uptake of hypoxia-sensitive PET tracers is dependent on tissue transport properties as well as hypoxia [13, 14, 17, 18, 25]. In principle, dynamic PET modeling corrects for transport properties such as slow tissue diffusivity that can impede the uptake of tracer and reduce sensitivity to hypoxia when such features are co-localized with hypoxia in PET voxels. This is especially problematic since PET voxels are typically large enough [ ∼(4 mm)^3^] to include diverse cell populations, with widely varying pathology [26]. The quantity of primary interest in a compartment model analysis of dynamic PET imaging is the trapping rate *k*_3_, commonly believed to be sensitive to hypoxia via the underlying binding kinetics [12–14]. Static PET imaging is more feasible clinically, however, and it is often assumed that one can adopt static imaging in place of kinetic imaging when some appropriate uptake metric–SUV for FDG-PET or TBR for hypoxia-PET–is well-correlated with *k*_3_ [27, 28].

In this paper, we have investigated dynamic and static PET in 20 patients with pancreatic adenocarcinoma (PDAC) and found *k*_3_ values to be only modestly correlated with TBR. Using Patlak’s formula to analyze these correlations, we found that a highly variable distribution volume across patients was primarily responsible for the reduced correlations, consistent with recent findings of FMISO kinetics in head and neck tumours [25].

**Fig. 6:**
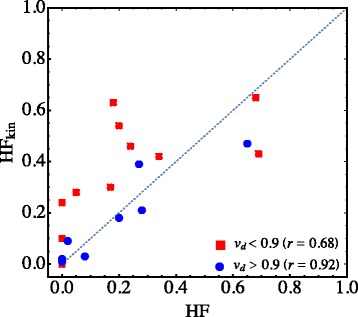
Impact of transport on calculation of hypoxic fraction. When *v*_*d*_>0.9, hypoxic fractions calculated from TBR>1.2 (HF) and *k*_b_>0.2 *h* ^−1^ (HF _kin_) are in substantial agreement. When *v*_*d*_<0.9, correlations are greatly diminished (*r*=0.68), with HF underestimating hypoxia

Correcting for the distribution volume, correlations were considerably stronger and the corrected tumour-to-blood ratio was *increased* (see Fig. 1). This shows that tracer uptake at 2 h in these patients is sensitive both to hypoxia and tissue transport properties (distribution volume), with the result that variability in tissue transport properties *reduces* the sensitivity of static PET imaging to hypoxia. Figure 6 compares hypoxic fractions in the twenty tumours calculated using: a.) the fraction of voxels for which TBR>1.2 and b.) the fraction of voxels for which *k*_b_>0.2 h ^−1^, a threshold chosen such that the two hypoxic fractions agree when transport effects are small (*v*_*d*_>0.9). When transport effects are substantial (*v*_*d*_<0.9), correlations between the two methods of calculating hypoxic fractions are greatly reduced (*r* goes from 0.92 to 0.68), with the TBR approach underreporting hypoxia on average.

At first glance, this would suggest that these tumours would benefit from dynamic PET imaging. The trapping rate was found to exhibit a strong dependence on the distribution volume, however, implying that *k*_3_ describes both the binding rate due to hypoxia as well as the rate of equilibration. A model was developed to explain this in which the extravascular tissue space was divided into two regions, one in which tracer rapidly achieved diffusive equilibration and one in which it equilibrated slowly. The population-averaged equilibration rate *k*_eq_≃(0.44±0.29) h ^−1^ in the latter region is consistent with our estimate in the Introduction of having either mucinous regions (on the order of tens to hundreds of microns in extent) where diffusivity is greatly slowed or micronecroses, smaller than a PET imaging voxel but larger than ∼ 500 *μ*m across.

The long equilibration time [1/*k*_eq_∼2.3 h] implied by this result means that unbound tracer will not equilibrate until well-after tracer injection, at times *t*≫1/*k*_eq_. At this time, the concentration of tracer in both the slow- and fast-equilibrating regions will approach that in blood and the effect of the distribution volume on TBR will vanish. Ideally, static hypoxia-PET imaging would be carried out when *t*≫1/*k*_eq_ in order to remove this sensitivity to transport. Unfortunately, the half-life of ^18^F is short and imaging times are typically restricted to be 3 h or less. (In our study, it was felt that accrual would be challenged by imaging patients past 2 h.)

**Fig. 7:**
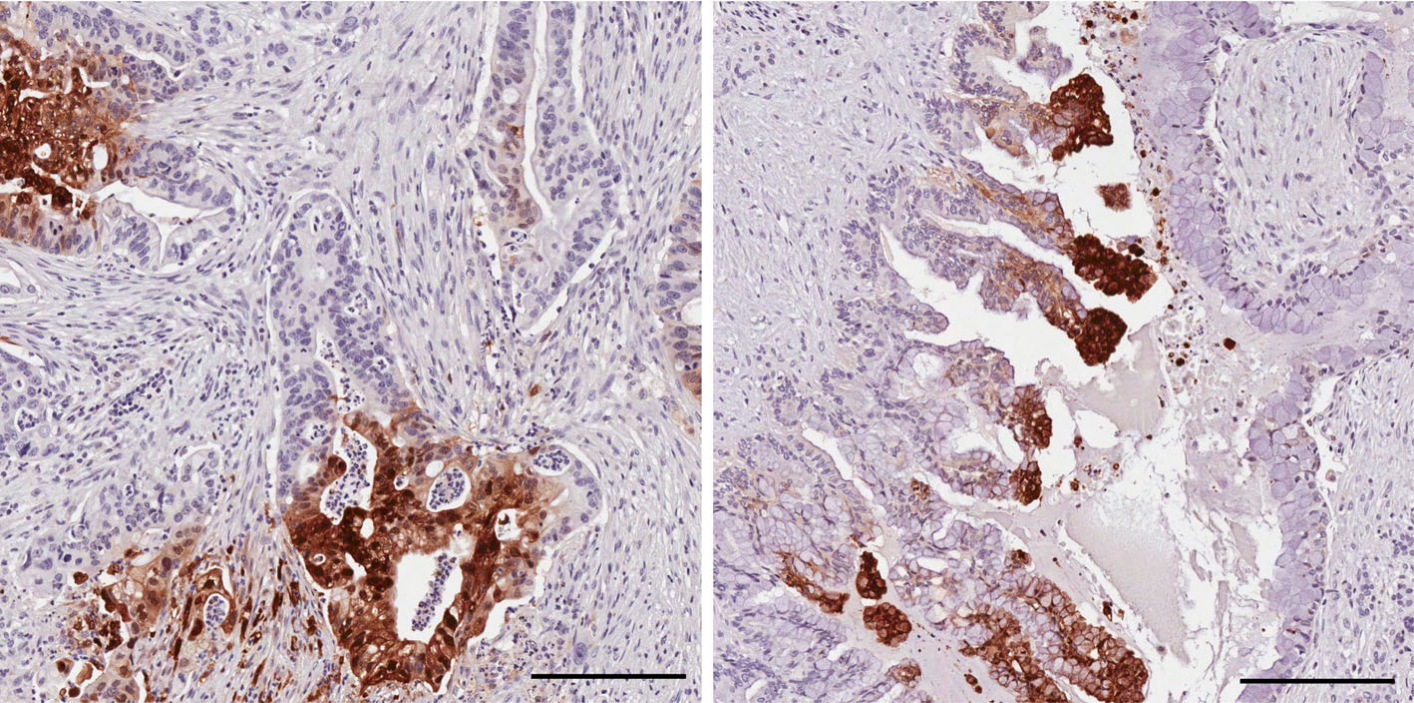
Resected histology slices from two patients (16 and 17 in Online Resource 1 (Additional file 1)), illustrating the hypothesized dependence of the distribution volume on mucin expression. The tumour on the left exhibits little mucin while that on the right exhibits abundant apical mucin. The average distribution volumes for these tumours are 0.92 and 0.76, respectively, representing above- and below average levels. The black scale bars in the lower-right hand corners of these plots indicates a length of 200 *μ*m; in comparison, a PET voxel is ∼ 4 mm across. Brown regions indicate staining for pimonidazole

If slow equilibration were due to necroses, *k*_1_–a measure of perfusion–would be correlated with *k*_eq_. No such correlations were found, leading us to hypothesize that mucous deposits comprised the regions of slow equilibration. Necroses are also rare in PDAC, whereas mucous gel-forming mucins are commonly over-expressed [9]. Amongst the twenty patients, the tumour volume fraction *v*_*d*_ in which tracer equilibrated rapidly varied from 0.68 to 1, with an average value of 0.85. This implies mucinous region volume fractions ranging from 0 to 30%, with an average value of 15%. Tumours were resected in four patients and examined by a pathologist [I.S.]. Although not a sufficient number to be able to definitively attribute the reduced distribution volume to mucous, the patients with the smallest and largest distribution volumes of this four exhibited significant and negligible mucin expression, respectively; see Fig. 7.

Our conclusion that equilibration is slow in parts of pancreatic tumours is not inconsistent with claims by us [21] and others [25] that *tumour-scale* equilibration rates are rapid. The characteristic equilibration rate in the fast-equilibrating regions can be approximated by *k*_1_ which, even for the hypo-perfused PDAC tumours studied in this work, was fast compared to *k*_b_ and *k*_eq_. The population average of the tumour-scale *k*_1_ values was ∼ 0.3 min ^−1^ [16]. Regions of slow-equilibration occupy a relatively small fraction of the tumours and hence, the tumour-scale equilibration rate is not strongly affected by these.

Although we have proposed a scheme to differentiate binding from equilibration, and hence, to quantify hypoxic status via the surrogate binding rate *k*_b_, the accuracy of this approach relies on the assumption that the variance in the equilibration rate is much smaller than the variance in the binding rate: $\left (\left.\sigma _{k_{\text {eq}}}\right /k_{\text {eq}}\right)\ll \left (\left.\sigma _{k_{\mathrm {b}}}\right /k_{\mathrm {b}}\right)$. Only then can we attribute the lowest few *k*_3_ values in each *v*_*d*_ bin to *K*_eq_ and not *k*_b_.

The fact that the estimated $\left (\left.\sigma _{k_{\text {eq}}}\right /k_{\text {eq}}\right)$ was only marginally smaller than $\left (\left.\sigma _{k_{\mathrm {b}}}\right /k_{\mathrm {b}}\right)$ means that our analysis did not completely distinguish equilibration and binding. In effectively assuming that the variance in the equilibration rate was zero, our analysis erred on the side of underestimating the equilibration rate and hence, overestimated the binding rate *k*_b_. At the same time, our scheme still represents an improvement over hypoxia quantification using *k*_3_ since *k*_3_ will always be larger than our estimated *k*_b_, which in turn is likely larger than the true *k*_b_. Full validation of our approach will rely on comparing our estimates of *k*_b_ and oxygen levels using other methods such as immunohistochemical staining of resected tumours. We plan on doing this in the future.

Beyond hypoxia quantification, dynamic PET imaging reveals additional information about tumour physiology that may prove to be clinically important [13, 14, 25, 29]. In our case, we have found that the distribution volume of FAZA (and likely all freely-diffusible PET tracers) quantifies the amount of mucous present in pancreatic tumours. Over-expression of the mucous gel-forming mucin MUC5AC in PDAC is prognostic for shorter survival time [30], greater metastatic potential [9, 31], and immune system avoidance [32]. We hypothesize that the distribution volume in other tumour sites will likewise provide complementary physiological information beyond hypoxic status.

A key question raised by this work is whether or not the tissue transport effects identified here confound hypoxia quantification using other hypoxia-PET tracers such as FMISO and in other tumour sites. The primary impediment to tracer equilibration is slow diffusivity. FAZA has been estimated to diffuse marginally faster than FMISO [7], and so the issues identified here should impact FMISO to a comparable degree. Indeed, similar effects as the ones reported here have arisen in FMISO imaging of pre-clinical tumour models [33], as well as clinical pharmacokinetic studies of head and neck tumours [17, 25]. In all cases, a variable distribution volume diminished correlations between TBR and *k*_3_. [The fact that *K*_*i*_=*v*_*d*_ *k*_3_ but not *k*_3_ was found to be well-correlated with TBR in Ref. [33] can be understood from Eq. (8): *K*_*i*_ removes the variance in TBR arising from *v*_*d*_ in the trapping term, but not the first two terms on the right-hand side of this equation.] In recent work, Grkovski et al. discuss the important role of the distribution volume in static PET hypoxia quantification and also report significant negative correlations between *k*_3_ and *v*_*d*_ [25]. The present work builds on these analyses by proposing a model in which *k*_3_ is sensitive both to hypoxia-induced binding as well as diffusive equilibration of un-bound tracer.

## Conclusions

The uptake of hypoxia-sensitive PET tracers in pancreatic tumours depends in a significant way on both tissue transport properties as well as the presence of hypoxia. Both dynamic- and static-PET based hypoxia surrogates— *k*_3_ and TBR—are affected by regions where diffusive equilibrium is achieved very slowly, over several hours. We have proposed a scheme to extract the hypoxia-sensitive tracer binding rate as well as the from dynamic PET data and proposed this as a novel hypoxia biomarker. Our results are of relevance for all hypoxia-PET tracers and any tumour site where transport of small-molecular weight agents is challenged.

## Additional files

Additional file 1 **Table S1.** (PDF 21 kb)

Additional file 2 Supplemental information. (PDF 100 kb)
