# Supplementary material for: Impact of tissue transport on PET hypoxia quantification in pancreatic tumours
Source: EJNMMI Res. 2017 Dec 22;7:101. doi: 10.1186/s13550-017-0347-3 (PMC5741574; doi:10.1186/s13550-017-0347-3)
Supplement: Supplementary file 1 — Table S1. (PDF 21 kb) [file 13550_2017_347_MOESM1_ESM.pdf]

Impact of tissue transport on PET hypoxia quantification in pancreatic tumours
Online Resource 1

patient	$r[k_3, \mathrm{TBR}(1\ \mathrm{hr})]$	$r[k_3, \mathrm{TBR}(2\ \mathrm{hrs})]$	$r[k_3, \mathrm{TBR}_{\mathrm{corrected}}(1\ \mathrm{hr})]$	$r[k_3, \mathrm{TBR}_{\mathrm{corrected}}(2\ \mathrm{hrs})]$	v_d
1	-0.20	-0.29	0.75	0.53	0.90
2	0.00	0.23	0.87	0.81	0.74
3	0.47	0.15	0.84	0.52	0.96
4	-0.32	-0.52	0.73	0.35	0.98
5	0.15	-0.23	0.77	0.44	0.97
6	-0.10	0.02	0.75	0.62	0.76
7	0.48	0.17	0.89	0.06	0.74
8	0.04	0.55	0.91	0.88	0.85
9	-0.12	0.02	0.84	0.88	0.80
10	0.14	0.17	0.94	0.93	0.86
11	0.20	0.37	0.73	0.77	0.98
12	-0.34	-0.05	0.64	0.83	0.94
13	0.36	0.59	0.92	0.68	0.77
14	0.56	0.45	0.84	0.65	1.00
15	-0.06	-0.25	0.87	0.30	0.68
16	0.20	-0.40	0.71	-0.08	0.92
17	0.45	-0.15	0.71	0.22	0.76
18	-0.58	-0.56	0.39	0.50	0.84
19	0.72	0.01	0.92	0.50	0.79
20	0.16	0.01	0.85	0.72	0.79
**AVERAGE**	**0.10**	**0.01**	**0.80**	**0.52**	**0.85**

TABLE S1: Pearson correlation coefficients r between the voxel-scale FAZA trapping rate k_3 and the tumour-to-blood ratio (TBR) and corrected $\mathrm{TBR}_{\mathrm{corrected}}$ tumour-to-blood ratios calculated from compartment modelling of the two- and one-hour data sets. Also shown are the average of the voxel-scale distribution volumes in each tumour. Population averages over all patients are shown at the bottom in bold font.
