# Supplementary material for: Impact of tissue transport on PET hypoxia quantification in pancreatic tumours
Source: EJNMMI Res. 2017 Dec 22;7:101. doi: 10.1186/s13550-017-0347-3 (PMC5741574; doi:10.1186/s13550-017-0347-3)
Supplement: Supplementary file 2 — Supplemental information. (PDF 100 kb) [file 13550_2017_347_MOESM2_ESM.pdf]

Impact of tissue transport on PET hypoxia quantification in pancreatic tumours
Online Resource 2

SENSITIVITY ANALYSIS OF THE FAZA BINDING AND EQUILIBRATION RATES

Here we assess the impact on the choice of threshold M used to distinguish binding from equilibrium using Eq. (19) in the main text. $M = 1$ would have been the appropriate choice if our data was noise-free. This would pick out the lowest value of k_3 in each v_d bin and associate it with the equilibration contribution $K_{\mathrm{eq}} = k_{\mathrm{eq}}(1 - v_d)/v_d$ to the trapping rate k_3. On the other hand, there is noise in our data arising from intrinsic PET noise as well as uncertainties associated with our model fitting. For this reason, we must choose a value for the cutoff that is greater than one. Increasing M beyond one, the estimate of k_{eq} is expected to increase, while that for k_{b} will decrease, since the sum $k_{\mathrm{b}} + K_{\mathrm{eq}} = k_3$ is fixed. When M is equal to the bin size $N_b = 10$, k_{b} will be zero since no values of k_3 are apportioned to binding.

In Fig. 1, we show the effect of varying M between one and $N_b = 10$ on the estimated values of k_{eq} and k_{b} for three patients. Patients 1 and 2 are the same as used in Fig. 2 in the main text. They represent normoxic and modestly hypoxic tumours, respectively, as estimated by the binding rate. We also show the sensitivity analysis for patient 13, since this was the most hypoxic tumour in our study. As expected, K_{eq} mostly increases with increasing M while k_{b} decreases nearly linearly. Assuming as much, the relative error for the binding rate is

$$\frac{\Delta k_{\mathrm{b}}}{k_{\mathrm{b}}} \simeq \frac{(\Delta M)}{N_b - 4} \tag{1}$$

for the threshold choice $M = 4$. Here, k_{b} is defined as the value of the binding rate determined for this choice of threshold and ΔM is the estimated error on the threshold choice. If the latter is ± 1, the relative error on the binding rate is 33%.

The choice of bin size N_b is not important for our sensitivity analysis. The bin size must be substantially larger than one to absorb statistical fluctuations, while at the same time, be much smaller than the total number of voxels N in order to deduce the v_d-dependence of k_3. As long both these conditions are satisfied, the above results hold for any choice of N_b, but with the threshold choice M scaled to accommodate the new choice; see Eq. (20) in the main text.

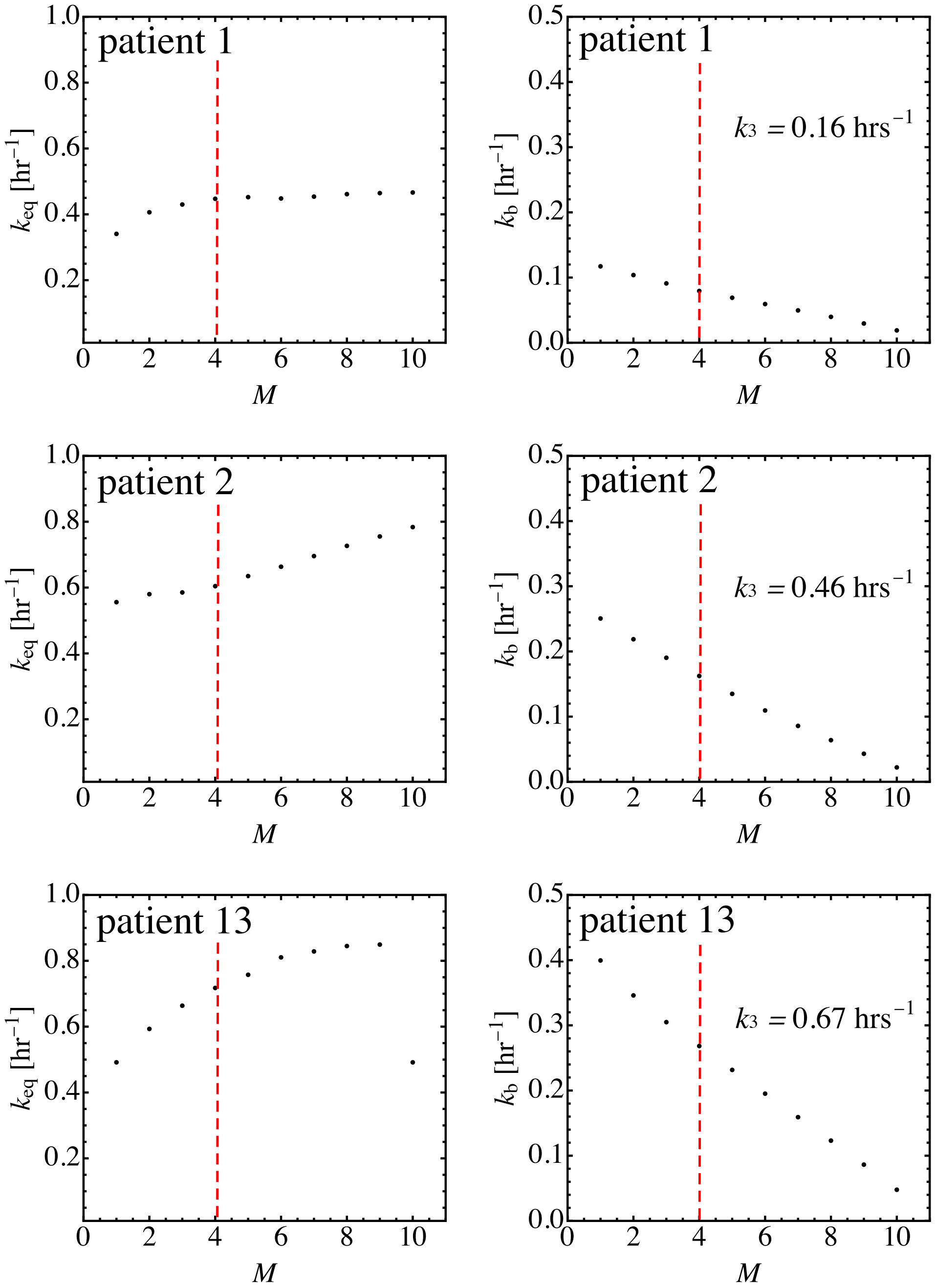

FIG. 1: Effect of threshold size M on the average of the voxel-scale equilibration ($k_{\rm eq}$) and binding ($k_{\rm b}$) rates for three different patients. The vertical dashed red line indicates our choice of threshold, $M = 4$. Patient 1 (top) represents a tumour of low hypoxia as determined by $k_{\rm b}$; Patient 13 (bottom) was the most hypoxic tumour studied. The average trapping rate values k_3 in each of the tumours are shown.
